# Photocatalytic Activity of N-Doped ZrO_2_ Thin Films Determined by Direct and Indirect Irradiation

**DOI:** 10.3390/ma16175901

**Published:** 2023-08-29

**Authors:** Carmen Mita, Nicoleta Cornei, Mariana Frenti, Georgiana Bulai, Marius Dobromir, Vasile Tiron, Aleksandr S. Doroshkevich, Diana Mardare

**Affiliations:** 1 Faculty of Chemistry, “Alexandru Ioan Cuza” University of Iasi, 11 Carol I Blvd., 700506 Iasi, Romania; cmita@uaic.ro; 2 Faculty of Physics, “Alexandru Ioan Cuza” University of Iasi, 11 Carol I Blvd., 700506 Iasi, Romania; marianafrenti2009@gmail.com; 3 Integrated Center of Environmental Science Studies in the North-Eastern Development Region—CERNESIM, Department of Exact and Natural Science, Institute of Interdisciplinary Research, “Alexandru Ioan Cuza” University of Iasi, 11 Carol I Blvd., 700506 Iasi, Romania; georgiana.bulai@uaic.ro; 4 Research Center on Advanced Materials and Technologies, Department of Exact and Natural Sciences, Institute of Interdisciplinary Research, “Alexandru Ioan Cuza” University of Iasi, 700506 Iasi, Romania; marius.dobromir@uaic.ro (M.D.); vasile.tiron@uaic.ro (V.T.); 5 Joint Institute for Nuclear Research, Str. Joliot-Curie, 6, Dubna 141980, Russia; doroh@jinr.ru

**Keywords:** N-doped ZrO_2_, HiPIMS, hydrophilicity, photocatalysis, RhB

## Abstract

In this paper, we investigate the decomposition of a toxic organic compound, Rhodamine B, by the photocatalytic activities of undoped and nitrogen-doped ZrO_2_ thin films, deposited using the HiPIMS technique. The investigation was performed in the presence and in the absence of H_2_O_2_, for two types of experimental arrangements: the irradiation of the films, followed by dipping them in the Rhodamine B solutions, and the irradiation of the films dipped in the solution. The two situations were named “direct irradiation” and “indirect irradiation”, respectively. Methods like XRD, AFM, XPS, DRS, water/film surface contact angle, and spectrophotometry were used to obtain information on the films’ structure, surface morphology, elemental composition of the films surface, optical band gap, hydrophilicity, and photocatalytic activity, respectively. All these properties were described and correlated. By N-doping ZrO_2_, the films become absorbent in the visible domain, so that the solar light could be efficiently used; the films’ hydrophilic properties improve, which is an important fact in self-cleaning applications; and the films’ photocatalytic activity for the decomposition of Rhodamine B becomes better. The addition of hydrogen peroxide acted as an inhibitor for all systems and not as an accelerator of the photocatalytic reactions as expected.

## 1. Introduction

Nowadays, the current global population growth and widespread industrialization are major factors contributing to significant environmental pollution caused by numerous hazardous wastes and organic contaminants. The ongoing release of organic pollutants, including dyes, into water resources leads to water pollution, posing risks to both human health and the aquatic ecosystems [1,2]. According to the Lancet Commission on Pollution and Health, approximately 1.8 million deaths worldwide are attributed to waterborne diseases [3]. These diseases are caused by a range of pollutants, including inorganic and organic compounds, nutrients, pathogens, and more. In this respect, organic pollutants play a significant role due to their prolonged persistence in wastewater. For these reasons, it is important to find solutions for their degradation. Photocatalysis is an effective oxidation process used in depollution and is recognized as an attractive “green” treatment. In this way, an important direction is to study the properties of the materials used as photocatalysts, in order to find the most performant ones.

Due to challenges in recycling and to minimize the need for extensive catalyst recovery, catalysts in thin-film forms have gained significant attention over powders. The films offer several advantages that make them promising catalysts for water treatment. One key advantage of the thin films is their high surface-area-to-volume ratio, which provides a higher number of active sites for photocatalytic reactions. This increased surface area allows for enhanced interaction between the catalyst and the target pollutants in water, leading to improved efficiency in the degradation of organic contaminants. Another advantage is the ease of fabrication and control over the film’s properties. Thin films can be deposited using various techniques, such as chemical vapor deposition, sputtering, spin-coating, etc., allowing for precise control over the film’s thickness, composition, and morphology [1,4,5]. Therefore, the study of the thin film’s properties used in the decomposition of organic pollutants through photocatalysis represents an important research direction and is the purpose of this paper. The chosen investigated film is zirconium dioxide, an environmentally friendly material, which is less studied in this form.

ZrO_2_ (zirconia) is a transition metal oxide that exists in three stable crystal structures: monoclinic, tetragonal, and cubic. The monoclinic form, also known as baddeleyite, is the room-temperature phase and is typically found with hydroxyl groups on its surface. Tetragonal and cubic ZrO_2_ become stable at temperatures above 1473 °C and 2673 °C, respectively. Due to its favorable chemical and physical characteristics, such as high chemical and thermal stability, high corrosion resistance, low thermal conductivity, high strength, and high transparency in the near infrared and visible domains, zirconia finds applications in various fields: oxygen sensors, fuel cells, catalysts, catalytic supports, high dielectric material for large-scale integrated circuits, gate dielectric in metal oxide semiconductors, etc. [2,6,7]. Regarding the use of this material as a photocatalyst, an important disadvantage is its large band gap energy (3.25–5.10 eV, as a function of preparation method), as reported by Navio et al. [8], for ZrO_2_ both in powder and thin-film forms. The high value of the optical band gap means that this material cannot efficiently use the sunlight because only 2–5% of solar terrestrial radiation is UV, while about 45% is in the visible range, depending on the geographic region, cloud cover, or pollution [9,10].

Different attempts were made to obtain efficient visible-light-driven photocatalysts (metal oxides like TiO_2_, perovskites, etc.) [11,12]. Some studies have demonstrated that the incorporation of nitrogen can effectively enhance the efficiency of ZrO_2_-based catalytic systems, making it absorbent in the visible domain [6,13,14].

In this paper, we have compared the photocatalytic decomposition of a very toxic organic compound, rhodamine B (RhB) [5], by using both undoped and N-doped ZrO_2_ thin films deposited on ITO/glass substrates, with and without a co-catalyst, namely H_2_O_2_. The investigation was performed for two situations: the irradiation of the films, followed by dipping them in the RhB solutions where the photocatalytic reactions take place, and the irradiation of the films dipped in the solution. We have named the two situations “direct irradiation” and “indirect irradiation”, respectively.

## 2. Materials and Methods

High-power impulse magnetron sputtering (HiPIMS) was used to deposit undoped (UDZ) and N-doped ZrO_2_ (NDZ) films (20 × 10 mm^2^) on unheated ITO/glass substrates. The deposition conditions were presented in a previous work [5]. Briefly, the total pressure was kept at 0.8 Pa, and a gas mixture consisting of Ar (50 sccm) and O_2_ (1 sccm) was used to obtain the UDZ film, while for the NDZ film, a gas mixture consisting of Ar (50 sccm), N_2_ (1 sccm), and O_2_ (0.1 sccm) was used. The repetition frequency was 800 Hz and 1250 Hz for NDZ and UDZ films, respectively.

A SHIMADZU 6000 diffractometer (CuK_α_ radiation, 40 kV, 30 mA) was used, in a θ–2θ geometry, to obtain the X-ray diffraction (XRD) patterns to acquire information on the crystalline structure of the films. The Scherrer formula [15] gives the average crystallite size values:

(1) $$D=\frac{0.9\lambda }{B_{1/2}\cos\theta }$$

where λ is the X-ray radiation wavelength (CuK_α_: 0.1518 nm), θ is the Bragg angle, and B_1/2_ is the width measured at half height of the diffraction peak.

The surface morphology of the thin films was analyzed with Atomic Force Microscopy (AFM) using a Scanning Probe Microscope (Solver PRO from NT-MDT, Moscow, Russia), in non-contact mode. To allow for quantitative comparison of the surface’s roughness across the investigated samples, all images were taken using the same cantilever (NSC21 from MikroMasch, with typical curvature radius of 10 nm, nominal spring constant of 19.1 N/m, and free resonant frequency of 227.9 kHz) and the same laser position. Microscope controlling, data acquisition, and image analysis were performed using Nova software version 1.0.26.1443 from NT-MDT.

From the X-ray photoelectron spectroscopy (XPS) measurements, information on the elemental composition and the chemical state of the atomic species at the film’s surface were acquired. The instrument, a Physical Electronics-Ulvac (PHI 5000 VersaProbe), was equipped with a mono-chromated Al Kα X-ray source, 1486.6 eV, power of 25 W. A Shirley background and Gaussian peak shapes were used to fit the spectra for all the components.

Diffuse reflectance spectra (DRS) were acquired in the range 190–1100 nm, using a Camspec 501M spectrophotometer, provided with an integrated sphere, coated with BaSO_4_, against quartz as a blank. The diffuse reflectance spectra were transformed into absorption spectra with the Kubelka−Munk function, F(R_d_) [16,17]:

(2) $$F\left(R_{d}\right)=\frac{\alpha }{m}=\frac{{\left(1-R_{d}\right)}^{2}}{2R_{d}}$$

where R_d_ is the diffuse reflectance, α is the absorption coefficient, and m is the scattering factor.

Knowing the dependence of the absorption coefficient, α, on the photon energy, hν, for the direct allowed transition, which dominates over the optical absorption in ZrO_2_ [16]:

(3) $${{\left(\alpha h\nu \right)}^{2}=A\left(h\nu -E_{g}\right)}_{}$$

where E_g_ is the optical band gap energy and A is a constant which does not depend on energy, an expression that contains the function F can be obtained:

(4) $${{{\left(Fh\nu \right)}^{2}=A_{0}\left(h\nu -E_{g}\right)}_{}}_{}$$

where A_0_ is a constant that contains the scattering factor m.

From the dependence ${\left(Fh\nu \right)}^{2}=f(h\nu )$, one can obtain the value of the optical bandgap, E_g_, by extrapolating the linear part of these dependences at Fhν=0.

A goniometer, Data Physics OCA 15EC, was the device used to study the wettability properties of the films. We worked with drop volumes of 500 nL deionized water to diminish both the evaporation effects and gravitational drop shape alteration. The films were photoactivated till the saturation of the photoactivation was achieved. The photoactivation was performed in environmental conditions (room temperature, 27 °C, and 60% environment humidity) with a high-pressure mercury lamp of 150 W, providing a flux of 1 mW/cm^2^ at the sample’s surface. The contact angles between the deionized water and the surface of the investigated films were measured at intervals of 5 min in 3 different locations on the film’s surface. Each final value, at each moment in time, was calculated as the average of the 3 values. At the end of the photoactivation experiment, the films were placed in a dark chamber, allowing their deactivation. The contact angles were measured every 12 h in the first 2 days, and, because the recovery was very slow, the measurements were collected every 24 h, for a total of 16 days.

The investigation of the films’ photocatalytic activity was performed by studying the RhB decomposition in the absence and in the presence of hydrogen peroxide (H_2_O_2_), using a 100 W visible LED lamp type 3C40B (HH-2507) provided with a cooling system. The preparation of RhB solution (3 mg/L) with and without the co-catalyst is presented in ref. [5]. We studied the films’ photocatalytic activities, considering two types of experiments: In the experiment that we have named “direct irradiation”, both films were irradiated for 15 min, and then each of the irradiated films was dipped for 20 min in a quartz cuvette containing 5 mL RhB solution, with the absorbance spectra being acquired from 5 to 5 min. The experiment continued by repeating the procedure until the difference between the absorbances was about 1%. To the best of our knowledge, there has been no attempt yet to use such an experiment to study the photocatalytic efficiency of a film. In the other experiment, named “indirect irradiation”, the films were dipped (with the face up) in 10 mL RhB solution at the bottom of the quartz cuvettes; the distance from the lamp to the film’s surface was fixed at 10 cm. The spectra were recorded every 20 min, until the constancy of the absorbance was obtained. Before irradiation, the systems (RhB solution and photocatalyst–RhB solution) were kept for one hour in dark conditions in order to ensure the adsorption–desorption equilibrium of the systems. In both experiments, the dye concentration was determined by measuring the absorption spectra with a Camspec 501M spectrophotometer at 553 nm (the specific maximum absorption wavelength from the visible domain for RhB).

In order to follow the evolution of the reaction mechanisms, in the first step, C/C_0_ vs. t^0.5^ was plotted. The rate constant values were calculated separately for each linear domain. For a better determination of the specific mechanism of RhB photodegradation, the reaction data were tested using pseudo-half-, pseudo-first-, and pseudo-second-order kinetic models. The relevant type kinetic was selected by applying the higher R^2^ correlation coefficient criteria.

The films’ stability and reusability were assessed by investigating their performance in four consecutive cycles. After each photodegradation reaction, to remove any impurities, the film’s surface was washed using ethanol and water and dried in an oven at 70 °C for 2 h.

## 3. Results and Discussion

### 3.1. Structural and Surface Topography Analysis

**Figure 1:**
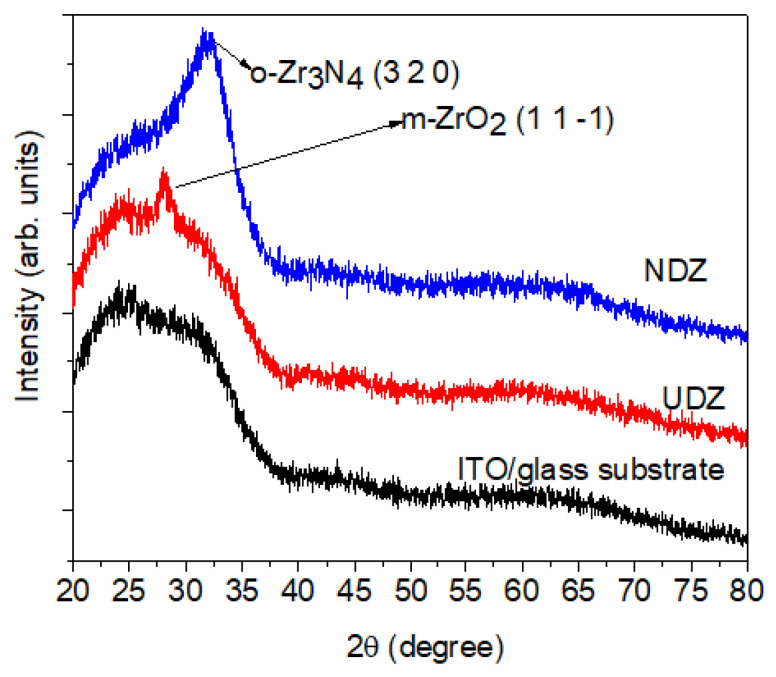
The X-ray diffraction patterns of undoped and N-doped ZrO_2_ films.

As we can see from the XRD patterns (Figure 1), the as-prepared films have a low crystallinity. However, for the undoped film, one can observe a main peak situated at 2θ = 28.10 deg., corresponding to the monoclinic structure, m-ZrO_2_(1 1 -1) (P121/c1 space group, JCPDS file no. 98-008-2212). For the N-doped film, a broad peak located around 2θ = 32.25 deg. is present; this is the main peak corresponding to the orthorhombic crystalline structure, o-Zr_3_N_4_(3 2 0) (Pna21 space group, JCPDS file no. 98-003-5747). The same phase transition was observed in our previous studies [5] for the undoped ZrO_2_ and N-doped ZrO_2_ films deposited on Si (100) substrates. There, the broad peak observed in the N-doped ZrO_2_ was assigned to both the tetragonal crystalline structure of zirconia (t-ZrO_2_ (0 1 1) at 2θ = 30.89 deg.) and to the orthorhombic crystalline structure of zirconia (o- Zr_3_N_4_, (3 2 0), at 2θ = 32.44 deg.).

The broadening of the peak o-Zr_3_N_4_ (3 2 0) can be attributed to multiple factors. One possible cause is the amorphous character of the film, where very small crystallites are present. Another explanation could be the superposition of several peaks predicted in this region for the o-Zr_3_N_4_ structure, as we have observed in our previous study [5] and explained by the asymmetry of the peak. These overlapping peaks can lead to a broader appearance in the X-ray diffraction pattern. Due to the limited number of observable diffraction peaks, it is challenging to definitively assign a specific crystalline structure to this sample. However, the available literature data suggest that the structure formed is highly similar to o-Zr_3_N_4_ but with the potential inclusion of oxygen atoms within the structure [18,19,20]. The presence of zirconium oxynitride in the NDZ film is confirmed by XPS data presented below, which show Zr-O, N-Zr-O, or N-Zr-OH bonds.

By calculating the crystallite size with the Scherrer equation, we observe a decrease in the crystallite size from 7.8 nm to 3.0 nm by nitrogen doping. This decrease may be due to several reasons. Firstly, the incorporation of nitrogen atoms into the ZrO_2_ lattice introduces lattice strain and distortion, which can inhibit the growth of larger crystallites. Secondly, nitrogen doping can act as a nucleation site for the formation of smaller and more dispersed crystallites. The same variation in crystallite size, depending on the doping nitrogen concentration, was also observed by Rawal et al. [21] and in our previous study [5].

**Figure 2:**
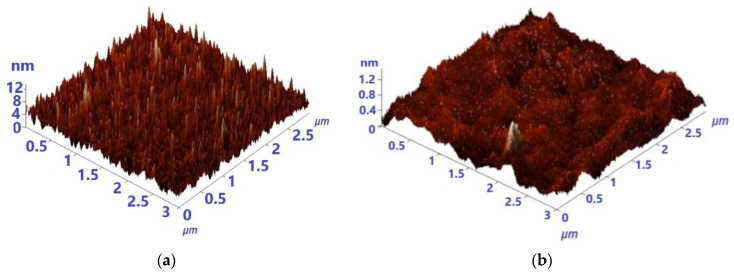
3D-AFM images for UDZ (**a**) and NDZ (**b**) films.

The investigated films are both very smooth, as seen from AFM investigations (Figure 2). This comes to an agreement with the commonly recognized fact that reactive sputtering determines the growth of smooth and uniform films [22,23]. The average roughness decreases as a result of doping, from 4.3 nm to 0.1 nm.

### 3.2. X-ray Photoelectron Spectroscopy Analysis

**Figure 3:**
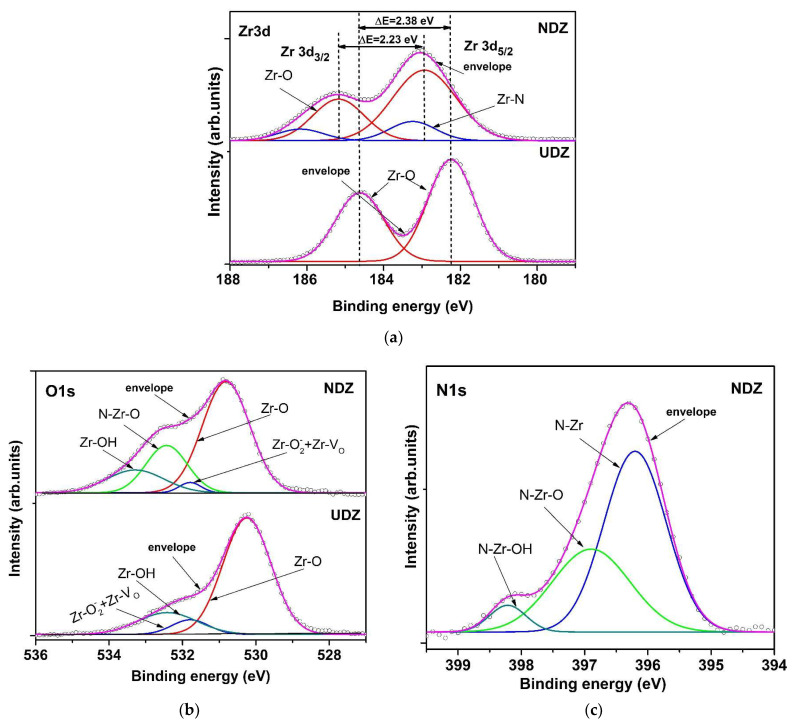
High-resolution spectra and peak fitting of Zr 3d (**a**), O 1s (**b**), and N 1s (**c**) of UDZ and NDZ films before photocatalysis.

The XPS spectra were recorded for the same films (UDZ and NDZ) before and after the photocatalytic experiments. The reason for this was to explain the effect of H_2_O_2_ especially on the photocatalytic properties of the directly irradiated films. Figure 3 shows the fitted spectra of Zr 3d, O 1s, and N 1s for UDZ and NDZ before photocatalysis.

The Zr 3d spectrum of UDZ exhibits two peaks, placed at 182.24 eV and 184.62 eV, assigned to Zr 3d_5/2_ and Zr 3d_3/2_ core level [24] with a spin orbit splitting of 2.38 eV (Figure 3a), which is very close to 2.4 eV of zirconia [25]. This indicates that zirconium ions are in the maximum oxidation state. For NDZ films, four fitting peaks were identified. The peaks located at 182.94 eV and 185.17 eV are assigned to Zr3d_5/2_-O and Zr3d_3/2_-O, and those located at 183.25 eV and 186.17 eV, having a 27.56% contribution, can be ascribed to Zr3d_5/2_-N and Zr3d_3/2_-N [26] (Figure 3a).

The split of Zr 3d-O main peaks suggests that the nitrogen atoms are incorporated into the ZrO_2_ lattice. The N incorporation induced the shift of Zr-O bond peaks to higher binding energies, with a simultaneous decrease in spin orbit splitting at 2.23 eV, which suggests that the oxidation state of zirconium is conserved, but its electronic density statisticaly decreases due to an increase in the number of anionic vacancies and the shift of the Fermi level to higher energy.

The O 1s XPS spectra are depicted in Figure 3b and consist of three peaks for UDZ and four peaks for NDZ samples. The main peak, located at 530.24 eV for UDZ and 530.81 eV for NDZ, corresponds to the oxygen lattice, having a contribution of 75.40% and 60.23%, respectively, of the total area of O 1s. The second peak, located at 531.79 eV for both samples, was assigned to oxygen vacancies (V_O_) and to Zr-$O_{2}^{-}$ bonds formed by the oxygen adsorption [27]. By nitrogen doping, a decrease in oxygen vacancies contribution from 7.23% to 2.57% of the total area of O 1s can be observed. For the NDZ sample, the peak located at 532.43 eV was assigned to the O-Zr-N bond with about a 22% contribution. Oxygen, due to presence of moisture, also forms the OH groups identified by their binding energy peaks centered at 532.1 eV for UDZ and 533.28 eV for NDZ. The presence of nitrogen in the NDZ sample determined a slight decrease in the electronic density in the O 1s level; this can be associated with a decrease in the number of oxygen vacancies which are compensated for by the increase in the OH and V_O_ areas ratio from 2.80 to 5.79. This observation explains the better hydrophilic properties of nitrogen-doped thin film.

Figure 3c presents the high-resolution XPS spectrum of N 1s for the NDZ film. There are three kinds of surface nitrogen species, having binding energies at 396.20 eV, 396.90 eV, and 398.22 eV, which can be assigned to the formation of Zr-N (Zr_3_N_4,_ nitride) [28], O-Zr-N, and HO-Zr-N (ZrO_2-x_N_y_, oxynitride) bonds, respectively [26]. The higher binding energy of N 1s from the oxynitride lattice is a consequence of both N 2p defect states and the more positive charge of nitrogen induced by the higher electronegative oxygen [29].

**Figure 4:**
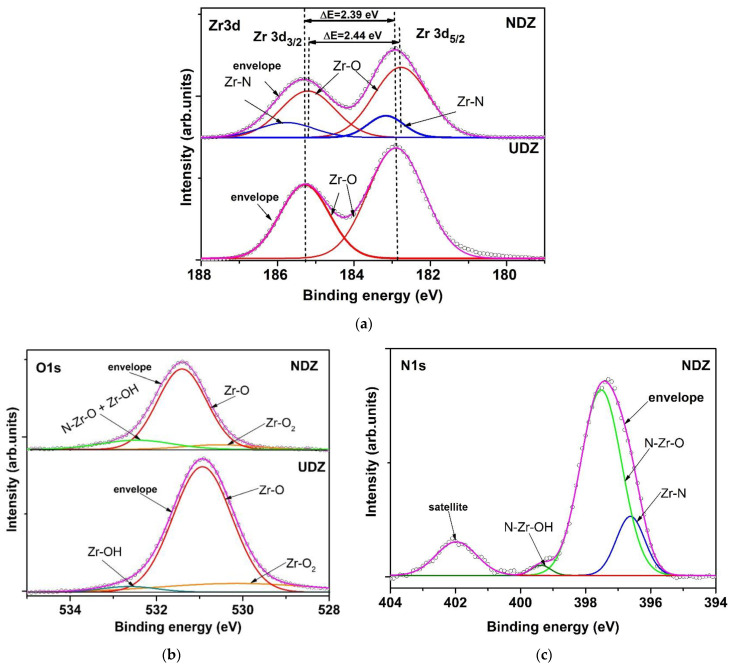
High-resolution spectra and peak fitting of Zr 3d (**a**), O 1s (**b**), and N 1s (**c**) of UDZ and NDZ films after photocatalytic experiments.

As will be seen in the section devoted to photocatalytic experiments, the presence of hydrogen peroxide inhibits the photocatalytic reactions and does not accelerate them as expected. To explain this behavior, the XPS spectra after photocatalysis were studied (Figure 4).

The XPS spectra of Zr 3d before and after photocatalysis are similar to both films but with a particularly positive core-level shift for UDZ and a negative shift for NDZ in the second case (Figure 4a). The Zr 3d signal consists of two components for UDZ and four components for NDZ. The Zr 3d_5/2_ and Zr 3d_3/2_ peaks located at 182.89 eV and 185.28 eV for UDZ and 182.76 eV and 185.20 eV correspond to Zr-O bonds with a spin orbit splitting of 2.39 eV for UDZ and 2.44 eV for NDZ. The additional peaks identified in the NDZ Zr 3d XPS spectrum, placed at 183.16 eV and 185.77 eV, are assigned to Zr-N bonds, having a contribution of 20%, which is 7% lower than in the first case, before photocatalysis. This could be due to a leaching of a part of the nitrogen atoms into the aqueous solution or, more probably, to the increase in oxygen contribution.

As is shown in Figure 4b, the O 1s signals are almost symmetrical, with the main peaks being located at a binding energy of 530.94 eV for UDZ and 531.41 eV for NDZ. The extended secondary peaks, centered at 530.14 eV (14.55%) and 532.63 eV (3.40%) for UDZ and 530.52 eV (8.44%) and 532.46 eV (16.90%) for NDZ, correspond to Zr-O-O^−^ and Zr-OH that, in the case of NDZ, is superposed with the O-Zr-N contribution. The decrease in the O-Zr-N general contribution could be a consequence of the occupation of a large part of the oxygen/anionic vacancies by the oxygen atoms generated by the peroxide groups and/or partial oxidation of nitrogen atoms from both the nitride and oxynitride lattice. Regardless of the mechanism, the photocatalytic activity of both thin films will be diminished by the hydrogen peroxide addition, more evidently in the case of the “direct irradiation” method.

The above XPS analysis result is reinforced by the allure of the N 1s spectrum for NDZ film (Figure 4c). The two peaks, fitted at 396.62 and 397.97 eV [6], are assigned to Zr-N (15.48% contribution) and N-Zr-O (70.23%). The peak centered at 399.37 eV (1.80% contribution) can be assigned to N-Zr-OH bonds or, more probably, to Zr-N-O bonds [30]. Further, the additional satellite peak fitted at 401.97 eV originated from N-O bonds [31], which suggests that the electronic density of nitrogen atoms decreased, and they can be in different oxidation states, with a small part in the positive valence state, such as NO interstitial species (Figure 4c).

### 3.3. Optical Bandgap

**Figure 5:**
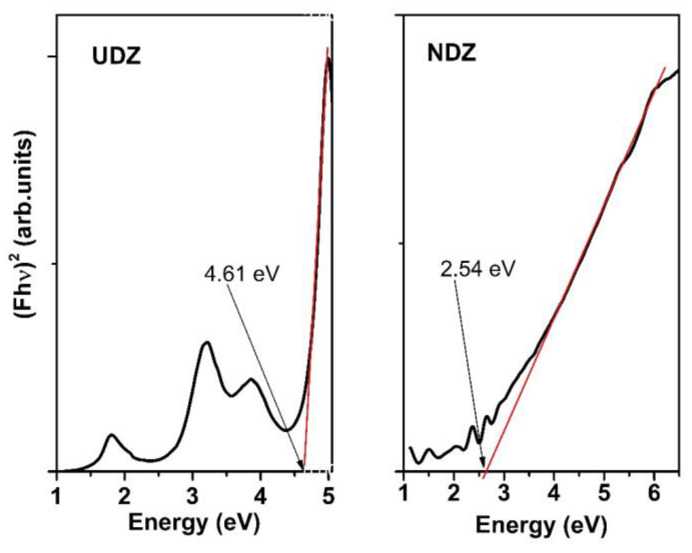
The plots ${\left(Fh\nu \right)}^{2}=f(h\nu )$ (calculated with the Kubelka–Munk function from DRS), considering that the direct allowed transitions dominate over the optical absorption.

As we have already mentioned, it is important for photocatalytic materials to be absorbent in the visible region of the solar spectrum. Since ZrO_2_, as a pure material, is absorbent in UV, we have investigated the possibility of extending the optical absorption edge to the visible domain. We have succeeded by using nitrogen doping. So, as can be seen from Figure 5, the optical bandgap, E_g_, decreases from 4.61 eV for the undoped film to 2.54 eV for N-doped film. The values of E_g_ were obtained by considering that the direct allowed transitions dominate over the optical absorption. For the linear dependence part in the plots ${\left(Fh\nu \right)}^{2}=f(h\nu )$, the correlation coefficients were fairly good (0.9970 for UDZ and 0.9876 for NDZ films). The data were fitted also for indirect allowed transitions, and the obtained E_g_ values were not satisfactory, being below 1 eV (in infrared domain) for both samples. So, we confirm once more the literature data, which state that the direct allowed transitions are the dominant ones in zirconia.

In the dependence ${\left(Fh\nu \right)}^{2}=f(h\nu )$ of the UDZ film, one can observe three additional smaller bands in the range 1.1–4.0 eV, two in the visible and one in the UV region of the spectrum. They could be due to the oxygen defects and local distortions generated by oxygen vacancies which determined the differences between the electronic density in specific directions and the implicit appearance of a dipole moment allowed transition at lower energy. These could act as supplementary bands that facilitate the hopping of electrons from the valence band (VB) to the conduction band (CB), acting as a middle band and electron trap [16]. Through the prism of these suppositions, we can explain the behavior of the undoped zirconia samples under visible light irradiation. In the case of the NDZ thin film, the surface defects and the nitrogen atoms play a similar role, acting as electron traps, preventing the electron–hole pair recombination [32].

### 3.4. Hydrophilic Properties

**Figure 6:**
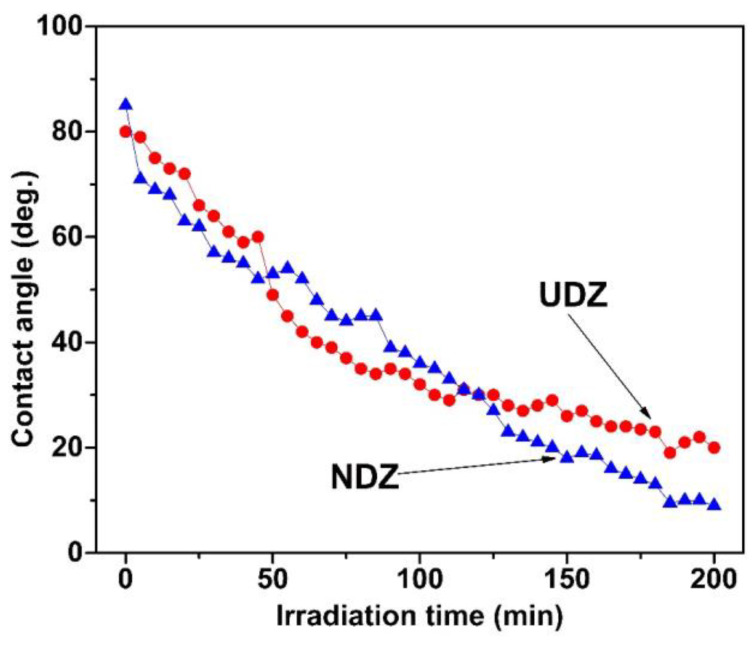
The time dependence of the contact angle between the deionized water and the films’ surface for UDZ and NDZ films during irradiation.

Figure 6 shows that the films have good hydrophilic properties, a fact explained by the presence of the OH groups on both of their surfaces, as revealed by XPS, with a special remark for the N-doped film, where, as mentioned above, we observed a higher OH/V_O_ area ratio.

The contact angle between the de-ionized water and the film’s surface presents a monotonical decrease during the irradiation. Even if the curves are quite close one to each other, we may affirm that the N-doped films possess better hydrophilic properties. The affirmation is sustained by the fact that, in the first 50 min of irradiation, there is a faster decrease in the contact angles corresponding to NDZ film, which finally, when approaching the irradiation saturation, reach superhydrophilicity (contact angles under 10 deg. [33]) with contact angles of 9 deg., while the undoped film reaches contact angle saturation values of only 20 deg. The lower crystallite size values in the N-doped film could favor a better adsorption of the molecules of water, which is reflected in a better hydrophilic activity and a higher photocatalytic activity, as will be shown below [22,34,35].

For hydrophilic surfaces (contact angles lower than 90 deg.), a rougher surface determines lower contact angles [34]. In our case, both films have very low roughness and high initial contact angles: 80 deg for UDZ and 85 deg for NDZ, which is in good agreement with Wenzel’s theory [34].

The incorporation of nitrogen into the ZrO_2_ lattice generates a number of additional vacancies in the anion sublattice:

(5) $$N_{2}+3O_{L}^{2-}\to 2N_{L}^{3-}+V_{O}+\frac{3}{2}O_{2}$$

where V_O_ represents the oxygen vacancies, and O_L_ and N_L_ are the lattice oxygen and nitrogen anions, respectively. This will lead to an increase in the number of defects and interactions with dioxygen and water molecules mobilized from the atmosphere. Then, a part of the adsorbed H_2_O molecules can interact with basic (more with O_L_ than N_L_) and acid Lewis centers (according to XPS analysis):

(6) $${Zr}^{4+}-O_{L}+H_{2}O↔{Zr}^{4+}-O_{L}\cdots H-OH$$

(7) $${Zr}^{4+}-V_{O}+H_{2}O↔{Zr}^{4+}\cdots OH_{2}$$

In addition, the process is thermodynamically and kinetically enhanced by the irradiation of the film. According to Rudakova et al. [36], the zirconia highly hydroxylated surface will be always hydrophilic, or even superhydrophilic, with the differences being given by the nature and concentration of the dopant.

**Figure 7:**
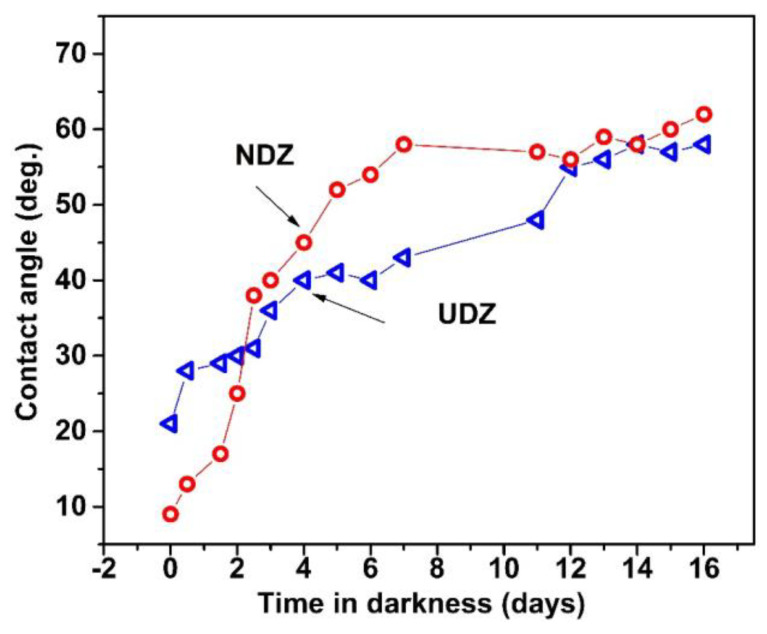
Contact angle vs. time dependences during the back-reaction regime for UDZ and NDZ films.

In the next stage, we investigated the recovery of the contact angle as a function of time by keeping the films in darkness. The increase in the contact angle for both films was evaluated for 16 days. Figure 7 shows that, even after 16 days, the films did not recover their initial contact angle, and the increases continue to be very slow. Though the contact angle recovery is slower for the UDZ film, both films reach almost the same value after 16 days.

The slow recovery of the contact angles for both films, meaning that the films remain activated for long periods of time, is important in self-cleaning applications. The highly hydrophilic surfaces induced by irradiation determine the complete wettability of the surfaces if more water drops are sprayed on their surface. When dirt is present, it does not adhere to the surface but floats in the water film and can easily be removed. The more a surface is activated for a longer period of time, the more it can maintain its hydrophilic properties and the better self-cleaning properties it has [37].

The above mentioned property of the films gave us the idea of investigating their photocatalytic activity using the method named “direct irradiation”, where the films are directly irradiated and then dipped in the solution to perform the RhB photodecomposition.

### 3.5. Photocatalytic Activity

The photocatalytic performance of UDZ and NDZ films under visible light was evaluated by studying the degradation RhB in aqueous solution in the absence and in the presence of hydrogen peroxide. When testing the domains of the kinetic type predominance, all photocatalytic systems show one linear domain over the entire time interval, except those with H_2_O_2_ irradiated using the “direct” method, for which two linear domains were identified. Consequently, the rate constant values were calculated separately for each domain. The higher R^2^ values were obtained for the pseudo-first-order kinetic model.

The catalytic degradation efficiency, DE (%), and the apparent rate constants of the pseudo-first-order model, k_app_, were calculated with the following equations:

(8) $$DE\;\left(\%\right)=\frac{C_{0}-C_{f}}{C_{0}}\cdot 100$$

(9) $$ln\frac{C}{C_{0}}=-k_{app}\cdot t,$$

where t is the reaction time (min), and C_0_ (mg·L^−1^), C (mg·L^−1^), and C_f_ (mg·L^−1^) are the initial, at the time t, and the final concentrations of the solution, respectively. The apparent rate constant reflects the variation in the concentration of chromophore groups in time and not of all intermediates of oxidation processes.

**Figure 8:**
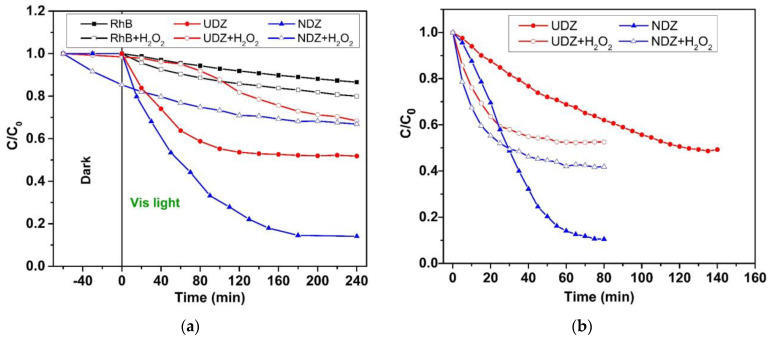
Photodegradation of RhB on UDZ and NDZ thin films using the (**a**) “indirect irradiation” method and the (**b**) “direct irradiation” method in the absence and in the presence of H_2_O_2_.

The photostability of RhB in aqueous solution in the absence and in the presence of H_2_O_2_ was tested at the beginning of the experiments. The degradation process proceeds slowly, with a degradation efficiency of about 15% in the absence and 20% in the presence of H_2_O_2_ after 240 min (Figure 8a). In the presence of UDZ and NDZ thin films, in the dark condition (Figure 8a), both catalysts have a sorption coefficient below 2%, except NDZ in the RhB-H_2_O_2_ solution (about 15%). In this particular case, it is possible for H_2_O_2_ to be activated by the anionic defects nearest the nitrogen atoms due to the natural affinity of nitrogen for oxygen and due to the reducing character of N(-III).

After 240 min, in visible light indirect irradiation, in the absence of H_2_O_2_, the photocatalytic system containing the NDZ film exhibits the highest DE (86%), while the UDZ film has a degradation efficiency of about 48%. The presence of H_2_O_2_ slowed down the rate of the reaction and decreased the DE for both catalysts at 42% and 33%, respectively. These results are in line with those previously obtained by us for nitrogen-doped zirconia thin film on Si(100) substrate [5] but in contradiction with the literature data on other photocatalysts [38,39]. A possible explanation was given above in the XPS data analysis.

Similar behavior was obtained for the photocatalytic systems “direct irradiated” but with higher DE in a shorter time (Figure 8b). The photocatalytic DE are as follows: NDZ (90%, 80 min), UDZ (51%, 140 min), NDZ-H_2_O_2_ (58%, 60 min), and UDZ-H_2_O_2_ (47%, 60 min). According to all these data, it can be observed that the photocatalytic activity of NDZ thin film is better than that of UDZ.

**Figure 9:**
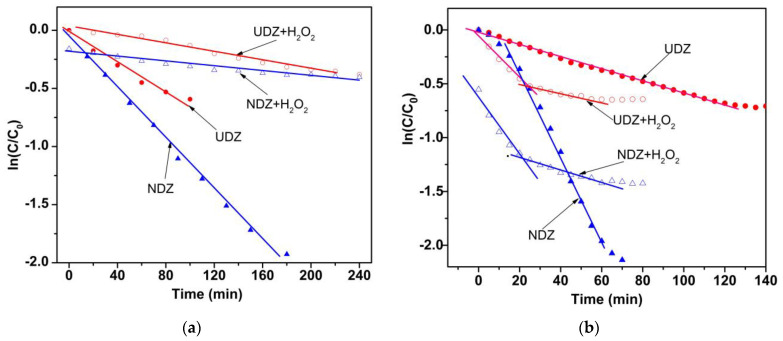
Kinetic plots for the degradation of RhB on UDZ and NDZ thin films using (**a**) indirect and (**b**) direct irradiation methods under Vis light.

In the following, the reaction kinetic was studied in all the cases. All reaction systems followed the pseudo-first-order kinetic. Kinetic plots are presented in Figure 9, and values of apparent rate constants are shown in Table 1.

As expected, the higher apparent rate constants were obtained for the NDZ film. However, the differences in the evolution of the reaction rate constant values for systems with H_2_O_2_ should be noted. For the “indirect irradiation” method, the photocatalytic reactions follow the same rate, with k_app_ values of 1.96 × 10^−3^ min^−1^ (UDZ) and 1.24 × 10^−3^ min^−1^ (NDZ), until a pseudo-equilibrium state is reached. In the case of the “direct irradiation” method, two stages with the same kinetic but with different values of the rate constants for RhB degradation were identified (Figure 9b). The initial rates, in the 0–30 min interval, were found to be 3 (UDZ) and 12 (NDZ) times higher than the reaction systems without H_2_O_2_. Then, after a 5–10 min transition period, the degradation rate became much slower, with close rate constant values of 3.52 × 10^−3^ min^−1^ (UDZ) and 3.70 × 10^−3^ min^−1^ (NDZ). In all photocatalytic reaction systems, H_2_O_2_ acted as an inhibitor, blocking a part of the active catalytic centers by the oxido-reduction process, in accordance with the XPS results. In conclusion, the addition of H_2_O_2_ acted as an inhibitor for all systems and not as an accelerator of the photocatalytic reactions, as stated in the majority of the papers in the literature.

It is widely known that in photocatalytic reaction systems, the reactive oxygen species $HO^{•}$ and $O_{2}^{-}$ play an essential role, with the hydroxyl radicals being considered the dominant reactive species in the oxidation reaction of organic substrates [40].

The possible mechanism to produce these highly reactive species is based on the photoactivation of ZrO_2_ and ZrO_2−x_N_y_ catalysts to generate electron–hole pairs through the transfer of electrons from the valence band (VB) to the conduction band (CB) [7,41]:

(10) $$ZrO_{2}+h\vartheta \to ZrO_{2}^{*}+h^{+}+e^{-}$$

(11) $$ZrO_{2-x}N_{y}+h\vartheta \to ZrO_{2-x}N_{y}^{*}+h^{+}+e^{-}$$

Then, the corresponding free radicals are generated by the following reactions:

(12) $$O_{2(ads)}+e^{-}\to O_{2}^{-}$$

(13) $$O_{2}^{-}+H_{2}O\to {HO}_{2}^{•}+HO^{-}$$

(14) $$H_{2}O+h^{+}\to H_{2}O^{+}\to H^{+}+HO^{•}$$

The photocatalytic activity mainly depends upon the separation of photogenerated hole–electron pairs and the transfer of electrons to dye towards anionic vacancies. The electrons tend to move to the surface of catalyst because of the electrostatic attraction with the positively charged catalyst surface. At the surface, the electrons will react with the adsorbed oxygen molecules and form oxidizing species such as $O_{2}^{-}$ and ${HO}_{2}^{•}$ (Equations (12) and (13)). The electron trap effect of nitrogen and crystalline lattice defects will slow down the recombination rate between electrons and holes. Thus, the generation of hydroxyl radicals by the holes is enhanced (Equation (14)). In the next stage, the reactive species will react with a reducing species, like RhB:

(15) $$RhB+HO\cdot \to CO_{2}+H_{2}O+other\;products$$

In addition, the holes themselves reaching the surface, could oxidize directly the adsorbed molecules of RhB:

(16) $$RhB+h^{+}\to RhB^{•+}\to intermediate\;products$$

The photoactivation of the catalysts in air (“direct irradiation” method), and not in aqueous solution, will increase the number of dioxygen adsorbed (due to partial pressure of O_2_) and activated (Equation (12)) on the film surface. This process could be enhanced by the air moisture (Equation (14)). All the active oxygen species, as well as holes, will react very quickly with RhB and water molecules upon contact with pollutant solution. The greater number of active species generated in a shorter time and the possibility of the additional reaction of RhB with the excess of holes (Equation (16)) could explain the higher reaction rate and yields upon direct irradiation.

In the photocatalytic systems with the addition of H_2_O_2_, in the first stage, the photocatalyst activates both H_2_O and the more reactive H_2_O_2_, which generate an increase in reactive oxygen species concentration, mainly hydroxyl radicals, according to Equations (14), (17) and (18):

(17) $$H_{2}O_{2}+e^{-}\to HO^{•}+HO^{-}$$

(18) $$H_{2}O_{2}+h^{+}\to HOO^{•}+H^{+}$$

The HO^•^ radicals will react preferentially with the RhB (more pronounced reducing agents) (Equation (14)), whereas, based on XPS analysis, the hydroperoxide radical (Equation (18)) can also react with a weaker $N_{L}^{3-}$ reducing agent on the surface of the catalyst, according to the following reactions:

(19) $$-N_{L}^{3-}+{\mathrm{HOO}}^{•}\to -N_{L}^{2-}-O-OH$$

(20) $$-N_{L}^{2-}-O-OH\to -N_{L}^{-}\ddot{-}O^{}+HO^{•}$$

or, in particular, to occupy some oxygen vacancies:

(21) $${Zr}^{4+}-V_{O}-{Zr}^{4+}+HOO^{•}+H^{+}\to 2{Zr}^{4+}-OH$$

These reactions can take place throughout the entirety of the photocatalytic process using both irradiation methods, with the probability and rates of the reactions 19–21 depending on the h^+^ concentration and the rate of ${HOO}^{•}$ radical generation, respectively. The excess of holes from the “direct irradiation” method will intensify the reactions 18–21 so that a larger number of active centers will be inactivated in a short time. At the same time, the excess of electrons will increase the ${HO}^{•}$ generation (Equation (17)) and, implicitly, the photodegradation rate of the dye (Equation (15)). In consequence, the decrease in active center concentration on the film’s surface could drive the increase in the probability of electron–hole recombination, which determines the decrease in the reaction rate, in our particular case, after 20–30 min of reaction time (Figure 9b). This could be a reasonable explanation for the mechanism of the whole process, evolution, and lower values of the apparent rate constants for RhB photodegradation in the presence of H_2_O_2_.

Since the films are very plane, the surface roughness, even slightly higher for the undoped film, is not a decisive factor that positively influences its photocatalytic performance.

Due to the enhanced photocatalytic activity in the visible range of NDZ compared to UDZ films, their applicability can be extended to the degradation of other organic pollutants, such as Methylene Blue, Amaranth, Indigo Carmine, etc., as evidenced by other studies [6,30,42]. Our investigation focused on Rhodamine B due to its specific characteristics but acknowledges that the broader applicability of N-doped ZrO_2_ thin films remains a topic for further exploration.

### 3.6. Stability, Reusability, and Thin Film Performance

**Figure 10:**
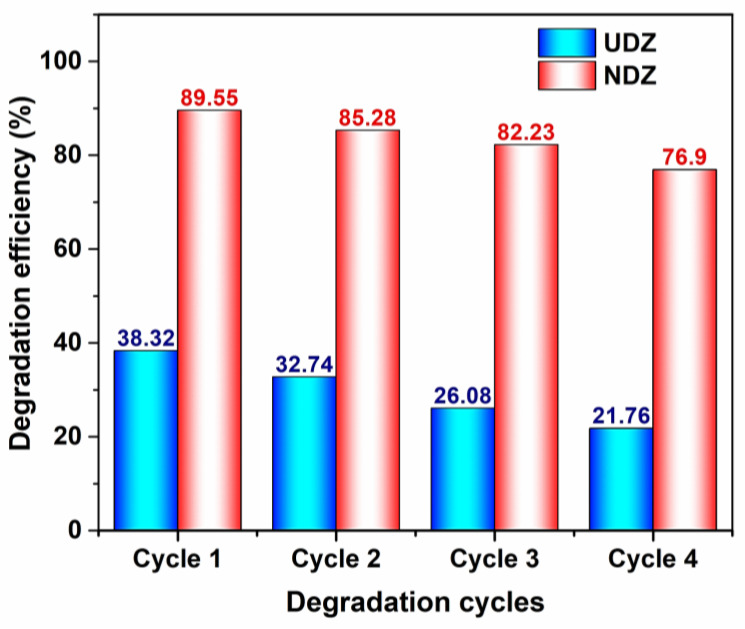
The photodegradation efficiency of RhB in the presence of UDZ and NDZ thin film after 80 min using “direct irradiation” methods.

The reusability of UDZ and NDZ catalysts was tested only for the photocatalytic degradation of RhB without H_2_O_2_ using the direct irradiation method. The optimum photocatalytic performance decreases after every cycle. The maximum degradation efficiencies (DE) were obtained for the NDZ film after 80 min of irradiation, while for UDZ, they were obtained after 160 min of irradiation. For a better comparison of the UDZ and NDZ films’ performances, Figure 10 shows the DE values recorded after 80 min of irradiation time. After the fourth cycle, the DE for NDZ decreased below 70%, and for UDZ, the DE decreased to about 38%.

It is hard to compare the photocatalytic efficiency of zirconia with those of other films, for instance with that of the titania films, because, even for the same material, the deposition method and the deposition parameters in each method have a high influence on their structure, morphology, and as a consequence, on their photocatalytic properties. Of course, the ratio between the catalyst quantity and the organic dye solution, the type and concentration of the dye solution, the lamp power, etc., lead to different conclusions in this sense. So, comparing different materials’ decomposition power is a challenge. Even so, in Table 2, we have conducted a comparison of the photodegradation efficiencies of our zirconia films and those of titania films under diverse experimental conditions.

From the examples above (Table 2), one can see higher photodegradation efficiencies for N-ZrO_2_/ITO.

## 4. Conclusions

Undoped and nitrogen-doped zirconia thin films were deposited on ITO/glass substrates using high power impulse magnetron sputtering.

The aim of N-doping, to obtain a shift of the fundamental absorption edge from the UV to the visible domain, a property wanted in photocatalysis, was accomplished.

Both undoped and N-doped films have good hydrophilic properties, with a special remark for the N-doped ZrO_2_, which becomes superhydrophilic after 200 min of irradiation, reaching contact angles of 9 deg., as explained by the XPS and XRD results. The time of recovery of the initial contact angles for both films was higher than 16 days, a property desired in self-cleaning applications.

The N-doped film has better photocatalytic activity for RhB decomposition, as revealed by the photocatalytic experiments conducted in two experimental settings, called “direct irradiation” and “indirect irradiation”, the first showing better photocatalytic activity. All reaction systems followed the pseudo-first-order kinetic, having two stages in the case of direct irradiation.

The photocatalytic activity of both thin films is reduced by the addition of H_2_O_2_, more obviously in the case of the “direct irradiation” method, with the explanation being related to the XPS results and reactivity of nitrogen centers. The addition of hydrogen peroxide acted as an inhibitor for all systems and not as an accelerator of the photocatalytic reactions, as mentioned in the majority of the papers in the literature. This is important for the environment if we consider the treatment of wastewater.

## Figures and Tables

**Table 1:** The kinetic parameters of pseudo-first-order model for the degradation of RhB on UDZ and NDZ thin films without (**−**) and with (**+**) addition of H_2_O_2_ under visible light irradiation.

Film	Parameter	Irradiation Method				
		Indirect		Direct		
		−	+	−	+	+
UDZ	k_app (_min^−1^)	1.06 × 10^−2^	1.24 × 10^−3^	5.52 × 10^−3^	1.82 × 10^−2^	3.52 × 10^−3^
	R^2^	0.9657	0.9745	0.9944	0.9592	0.9772
NDZ	k_app (_min^−1^)	5.97 × 10^−3^	1.96 × 10^−3^	1.66 × 10^−2^	1.96 × 10^−2^	3.70 × 10^−3^
	R^2^	0.9845	0.9703	0.9899	0.9485	0.9405

**Table 2:** Comparison of the photodegradation efficiencies of the studied zirconia films and those of titania films for different experimental conditions.

Photocatalyst	Preparation Method	Active Surface of the Film	Light Source	Dye Concentration/Volume	Irradiation Time/Degradation Efficiency (%)	Ref.
ZrO_2_/ITO	HiPIMS *	20 × 10 mm^2^	100 W/visible LED lamp	3 mg·L^−1^ RhB/10 mL	240 min./48% (“indirect irradiation”)	This work
				3 mg·L^−1^ RhB/5 mL	140 min./51% (“direct irradiation”)	
N-ZrO_2_/ITO	HiPIMS **	20 × 10 mm^2^	100 W/visible LED lamp	3 mg·L^−1^ RhB/10 mL	240 min./86% (“indirect irradiation”)	This work
				3 mg·L^−1^ RhB/5 mL	80 min./90% (“direct irradiation”)	
TiO_2_/FTO	Hydrothermal synthesis/120 °C/10 h	35 × 35 mm^2^	300 W/Xenon lamp	5 mg·L^−1^ MB/100 mL	300 min./59%	
0.5%N-TiO_2_/FTO					300 min./95%	
1%N-TiO_2_/FTO					300 min./57%	[43]
2%N-TiO_2_/FTO					300 min./66%	
0.5%Fe_2_O_3_/TiO_2_/FTO	Hydrothermal nanorod/160 °C/2 h	20 × 20 mm^2^	10 W/365 nm LED	4.8 mg·L^−1^ RhB/20 mL	300 min./52%	[44]

* Gas mixture: Ar (50 sccm), O_2_ (1 sccm). ** Gas mixture: Ar (50 sccm), N_2_ (1 sccm), O_2_ (0.1 sccm).

## Data Availability

All data are available in the manuscript or upon request to the corresponding author.
